# Laparoscopy in Trauma: Can Brazil fit into the global trend?

**DOI:** 10.1590/0100-6991e-20233602EDIT01-en

**Published:** 2023-09-22

**Authors:** SERGIO HENRIQUE BASTOS DAMOUS, CARLOS AUGUSTO METIDIERI MENEGOZZO, LUIZ CARLOS VON-BAHTEN, EDIVALDO MASSAZO UTIYAMA

**Affiliations:** 1 - Hospital das Clínicas da Faculdade de Medicina da Universidade de São Paulo, Disciplina de Cirurgia Geral e Trauma - São Paulo - SP - Brasil; 2 - Pontífica Universidade Católica do Paraná, Cirurgia - Curitiba - PR - Brasil; 3 - Universidade Federal do Paraná, Cirurgia - Curitiba - PR - Brasil

## EDITORIAL

Two decades ago, laparoscopy inaugurated a new era in the treatment of trauma victims, allowing surgeons to diagnose intra-abdominal injuries in a minimally invasive way. However, the high incidence of unnoticed lesions was a reason for resistance to implementing the method as a routine^1^ ^-^ ^3^. With the improvement of video equipment, the greater experience of surgeons, the standardization of the exam for laparoscopy in trauma, and the adequate selection of patients, diagnostic laparoscopy was safely incorporated in the management of these patients, avoiding unnecessary laparotomies^4^ ^-^ ^8^. Once the need for therapeutic procedures is confirmed, laparotomy is still the routine access route.

The current discussion revolves around therapeutic laparoscopy, that is, the concrete possibility not only of diagnosing, but of treating traumatic injuries, even in the case of multiple lesions, of different degrees of complexity, both in penetrating and blunt trauma, the latter being even more challenging^9^ ^,^ ^10^. The relevance of the subject today can be proven by the growing number of scientific publications. In a search in the Pubmed database with the keywords “abdominal trauma” and “laparoscopy”, one finds 300 results from the last 20 years, 60% of which in the last 10 years (2013-2023) and 30% in the last five years (2018-2023). Restricting the search with the descriptors “abdominal trauma” and “therapeutic laparoscopy” renders 37 results in the last 20 years, 73% in the last 10 years (2013-2023) and 49% in the last five years (2018-2023).

In the ideal scenario, the care of trauma patients should be performed by an emergency surgeon trained to treat trauma. The discussion on how to train a trauma surgeon remains elusive. This specialist is often seen as a surgeon specializing in critical care and advanced clinical support. To safely insert the minimally invasive technique in trauma, there must be an association of the surgeon’s experience in caring for traumatized patients (multidisciplinary approach) and the technical qualification in advanced laparoscopic procedures, which are acquired in the elective or non-traumatic emergency settings^11^ ^-^ ^13^.

The emergency surgeon who does not work with elective surgeries has fewer opportunities to perform minimally invasive operations, reflecting in a lower technical capacity to perform advanced laparoscopic procedures. The skill in laparoscopy is divided into three groups of procedures: 1) Group 1 - Basic procedures (i.e., appendectomy and cholecystectomy); 2) Group 2 - Intermediate procedures (i.e., Gastric fundoplication, hernioplasty, gastrointestinal suture, sleeve gastrectomy, and splenectomy); and 3) Group 3 - Advanced procedures (i.e., esophagectomy, gastrectomy, colectomy, pancreatectomy, gastric bypass, and hepatectomy). The basic skills needed to perform laparoscopy in trauma are properly positioning the patient, handling the camera accurately, safely moving the organs in the abdominal cavity, inspecting intestinal loops, performing sutures, and applying hemostasis techniques^11^. These skills can be acquired by performing exercises in a box with a camera, using virtual reality simulators, or handling tissues in animal models (pigs)^13^. In trauma, the surgeon needs to have technical skills in advanced laparoscopy, as these are urgent situations in a hostile environment and require quick decision-making, the availability of adequate material also being paramount^10^ ^,^ ^12^ ^,^ ^14^.

In 2021, we carried out a survey sent by email to surgeons affiliated with the Brazilian College of Surgeons, to evaluate the use of minimally invasive surgery in the diagnosis and treatment of patients who are victims of abdominal trauma. Of the 114 who participated, only 8% worked in Trauma services, the most frequent work regime being the on-site general surgery shift (63%) in a public hospital (44%) in the Southeast region (59%). The predominant character of surgical activity is exercised through elective surgeries (63%). The most frequently mentioned reasons for not performing laparoscopic access in trauma were difficulty in accessing the material in the emergency room, lack of equipment in the service, and lack of trained staff.

Although the conversion rate pointed out by most respondents (29%) was < 10%, the reasons listed for conversion from laparoscopy were diverse, such as multiple organ injuries, complex visceral injuries, distension of the abdominal loops, and need for visceral resection, with risk of associated lesions. Such difficulties can be circumvented by the surgeon’s experience in advanced laparoscopic procedures if the patient remains hemodynamic stable. In a recent report from our group, we describe the barriers and challenges over 15 years to implement laparoscopic access in emergency surgeries (appendectomy). In addition to training the surgical team, constant investment in materials and standardization of feasible and safe techniques are essential, and only with the balance of such conditions was it possible to make laparoscopic appendectomy routine in a tertiary service of medical education in Brazil^15^.

The consecrated advantages of laparoscopy, such as shorter hospitalization time, early return to activities, and lower incidence of infection are also evidenced in the trauma context^2^ ^,^ ^4^ ^,^ ^16^ ^-^ ^18^. Prospective studies are still scarce, but retrospective observational studies have shown similar and encouraging results towards validating the role of laparoscopy in trauma, rendering this approach increasingly used for therapeutic purposes^7^ ^,^ ^9^ ^,^ ^14^ ^,^ ^16^ ^-^ ^19^. For the selection of eligible patients, contrast-enhanced computed tomography (CT) scan is a great ally for assessing vascular and retroperitoneal lesions, in addition to identifying associated ones. Mapping by CT allows adjusting and adapting the positioning of the patient and the trocars according to the location and type of injury, defining the best operative strategy and therefore enhancing the accuracy of laparoscopy in this scenario^13^ ^,^ ^20^ ^,^ ^21^.

There are several technical challenges to be overcome, such as difficulty in patient selection, lack of systematization in the abdominal cavity inventory, surprise effect of findings, number of associated injuries, hostile environment in the abdominal cavity (blood and contamination), and inadequate visualization of the retroperitoneum. There are also difficulties related to the availability of adequate equipment^9^ ^,^ ^10^ ^,^ ^13^. In recent years, studies have proven that laparoscopy in trauma is a safe and feasible procedure, its indication has grown, and the results are excellent. Therefore, it is necessary to structure emergency services in Brazil with the tripod trained surgeons, adequate selection of patients, and full-time availability of materials and subsidiary tests.
